# Centromere protein-A, an essential centromere protein, is a prognostic marker for relapse in estrogen receptor-positive breast cancer

**DOI:** 10.1186/bcr3181

**Published:** 2012-05-04

**Authors:** Susan L McGovern, Yuan Qi, Lajos Pusztai, William F Symmans, Thomas A Buchholz

**Affiliations:** 1 Department of Radiation Oncology, 1515 Holcombe Blvd., University of Texas MD Anderson Cancer Center, Houston, Texas, 77030, USA; 2 Department of Bioinformatics and Computational Biology, 1515 Holcombe Blvd., University of Texas MD Anderson Cancer Center, Houston, Texas, 77030, USA; 3 Department of Pathology, 1515 Holcombe Blvd., University of Texas MD Anderson Cancer Center, Houston, Texas, 77030, USA

## Abstract

**Introduction:**

Centromere protein A (CENP-A), an essential centromere protein, has been associated with high grade cancers. This study was undertaken to determine if CENP-A is a prognostic factor for breast cancer patients not receiving systemic therapy or predictive of response to tamoxifen or neoadjuvant chemotherapy.

**Methods:**

mRNA levels of CENP-A and CENP-B, a centromere protein that binds independently of CENP-A, were measured in breast cancer specimens from 484 patients receiving no systemic therapy, 276 patients receiving tamoxifen, and 233 patients treated with neoadjuvant chemotherapy. Associations between CENP-A, CENP-B, Ki-67, relapse, and chemotherapy response were determined.

**Results:**

CENP-A but not CENP-B was higher in estrogen receptor (ER)-negative tumors than ER-positive tumors and positively correlated with Ki-67 expression. Among patients with ER-positive disease who received no systemic therapy or tamoxifen, higher levels of CENP-A were associated with lower rates of 5-year distant relapse free survival (DRFS). On multivariate analyses including Ki-67, high CENP-A expression had a hazard ratio of 10.9 for relapse in patients with ER-positive disease not receiving systemic therapy (95% CI, 2.86 to 41.78; P = 0.00047) and 1.64 for patients with ER-positive disease receiving tamoxifen (95% CI, 0.99 to 2.71; P = 0.054). CENP-A was not an independent prognostic marker in ER-negative tumors. For both ER-positive and ER-negative tumors, CENP-A was not a significant independent predictor of chemotherapy response.

**Conclusions:**

CENP-A was a significant independent prognostic marker for patients with ER-positive breast cancer not treated with systemic therapy but had limited predictive value in tamoxifen treated patients and was not predictive of response to neoadjuvant chemotherapy.

## Introduction

Faithful chromosome segregation during cell division requires precise assembly of the kinetochore protein complex on centromeric chromatin [1]; aberrations in this process cause chromosomal instability and aneuploidy [2]. Because the DNA sequence of centromeres is not conserved, it is widely thought that the marker of centromere location is a protein, centromere protein-A (CENP-A). It is a 17 kDa variant of histone H3 and is found at all active centromeres [2,3]. Overexpression of CENP-A causes ectopic formation of multicentric chromosomes and functional kinetochores [4]. Inversely, depletion of CENP-A promotes apoptosis and induces cell cycle arrest [5,6].

Extending these observations, recent translational work has shown that CENP-A is elevated in tumor cells compared to normal [6,7]. Increased expression of CENP-A is associated with higher grade cancers [6,8] and increased invasiveness [8]. Consequently, CENP-A has been included in predictive genetic profiles in breast cancer [9,10]. These observations suggest that elevated CENP-A may be correlated with poorer patient outcomes.

Recent results also suggest that CENP-A is recruited to sites of DNA damage and may participate in repair of double strand DNA breaks [11]. Because of this possible role in DNA repair, elevated levels of CENP-A might promote resistance to chemotherapy.

CENP-A may also provide unique prognostic and predictive information in estrogen receptor (ER)-positive breast cancer. At least two different mechanisms for the influence of the ER on CENP-A are possible. In the first, the transcription factor, forkhead box protein M1 (FOXM1) is essential for transcription of CENP-A [12]. Recent work has shown that ER alpha regulates FOXM1 expression in breast cancer cell lines [13], suggesting a pathway for ER to modulate CENP-A levels. In a second possible mechanism, estrogen exposure increases expression of Aurora A kinase [14], which is required to phosphorylate CENP-A for proper kinetochore function at mitosis [15]. In ER-positive disease, there may be higher levels of phosphorylated CENP-A and more functional kinetochore complexes compared to ER-negative disease. Moreover, both of these potential mechanisms of estrogen-driven CENP-A modulation may result in greater sensitivity to tamoxifen in cells overexpressing CENP-A.

To further explore these hypotheses, we analyzed CENP-A as a prognostic and predictive biomarker in ER-positive and ER-negative breast cancer. The association of CENP-A with outcome was determined in two distinct groups of patients: 484 patients from two datasets of patients receiving no systemic therapy and 276 patients receiving tamoxifen alone. To determine if CENP-A predicted for response to chemotherapy, the association of CENP-A with the volume of residual disease was determined in 233 patients treated with neoadjuvant chemotherapy.

As a control, we compared CENP-A with centromere protein-B (CENP-B), a protein that localizes to the centromere independently of CENP-A [16] and remains at the centromere throughout the cell cycle [3]. Previous work has shown that in normal cells, almost all of the CENP-A co-localizes with CENP-B. In tumor cells overexpressing CENP-A, more than ten percent of CENP-A no longer co-localizes with CENP-B [7], suggesting that excess CENP-A binds at non-centromeric regions.

ER status has emerged as a determinant of genetic profiles of breast cancer [17]. The results described below explore the role of CENP-A as a prognostic and predictive marker in ER-positive and ER-negative breast cancer.

## Materials and methods

### Patient populations

**Figure 1:**
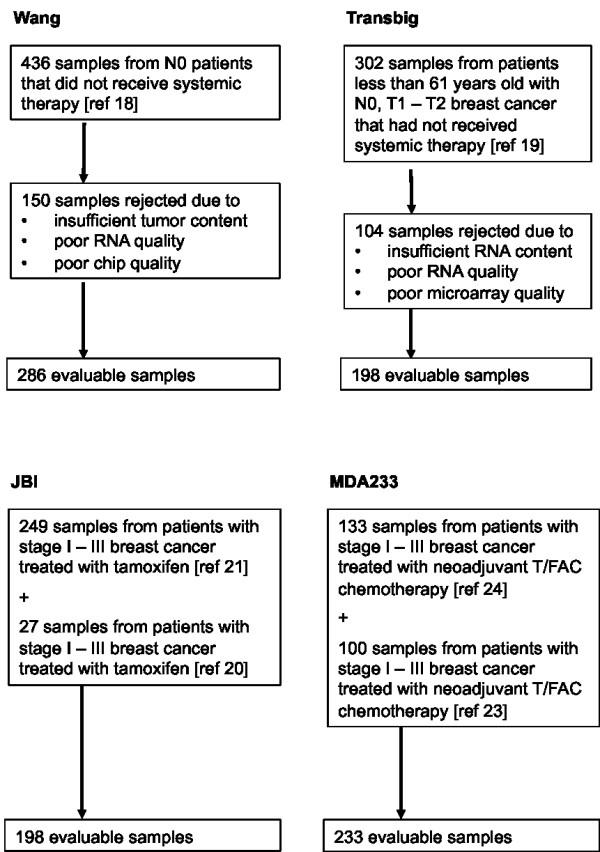
**Datasets used in this study**.

To determine the prognostic ability of CENP-A, we used two separate datasets of clinically node-negative patients who did not receive systemic therapy. The first is a cohort of 289 patients previously described by Wang, *et al. *[18] with gene expression data publicly available at the Gene Expression Omnibus (GEO) database [GEO:GSE2034]. The second is a series of 198 patients previously reported by the TRANSBIG consortium [19] with gene expression data available at the GEO database [GEO:GSE7390]. To evaluate the ability of CENP-A to predict response to tamoxifen, we used a dataset of 276 patients from the Institut Jules Bordet (JBI) [10,20,21]. Expression data are available at the GEO database [GEO:GSE2990]. Finally, to determine the value of CENP-A as a predictor of response to neoadjuvant chemotherapy, the MDA233 dataset of 233 patients treated with either weekly (80 mg/m^2 ^for 12 doses) or once every three weeks (225 mg/m^2 ^for four doses) paclitaxel followed by four cycles of 5-fluorouracil (500 mg/m^2^), doxorubicin (50 mg/m^2^), and cyclophosphamide (500 mg/m^2^; T/FAC) was used. Response to chemotherapy was categorized using the residual cancer burden (RCB) index based on residual disease at the time of surgery. Patients were categorized as having no (RCB = 0), minimal (RCB = 1), moderate (RCB = 2) or extensive (RCB = 3) residual disease as previously described [22]. Gene expression data from this cohort have been previously described [23,24] and are available at the MD Anderson Bioinformatics website [25]. For all four datasets, ER status was determined using ligand binding assay [18,21], EIA [18], or immunohistochemistry [18,19,24]. A summary of dataset properties is shown in Figure 1. Patient characteristics for all four datasets are shown in Table 1.

**Table 1:** Patient characteristics.

Dataset	Wang	TRANSBIG	JBI	MDA233
Patients (number)	286	198	276	233
Age (y)				
Median	Unknown	46	64	51
Range		24 - 60	40 - 88	26 - 79
T stage				
T1	0	102	107	23
T2	0	96	149	132
T3 or T4	0	0	8	78
Unknown	286	0	12	0
Tumor grade				
1	0	30	50	13
2	0	83	130	96
3	0	83	47	124
Unknown	286	2	49	0
Node status				
Positive	0	0	142	166
Negative	286	198	117	67
Unknown	0	0	17	0
ER status				
Positive	209	134	266	142
Negative	77	64	10	91
Unknown	0	0	0	0
PR status				
Positive	0	0	135	0
Negative	0	0	29	0
Unknown	286	198	112	233
Her2/neu status				
Positive	0	0	0	142
Negative	0	0	0	91
Unknown	286	198	276	0

ER, estrogen receptor; PR, progesterone receptor.

### Gene expression analysis

For all four datasets, mRNA expression levels were measured using the Affymetrix HG-U133A Gene Chip (Santa Clara, CA, USA) using standard procedures as previously described [18,19,21,24]. mRNA levels were normalized using the MAS5 method to a target intensity of 600 followed by log2-transformation [26]. After normalization and transformation, a one-point increase in log2-transformed value corresponds to a doubling of mRNA expression. The following Affymetrix U133A probe set identification codes were used: CENP-A, 204962_s_at; CENP-B, 212437_at; HER2, 216836_s_at; Ki-67, 212022_s_at; and ESR1, 205225_at.

### Statistical analysis

Correlations between CENP-A, CENP-B levels and ER status; CENP-A and RCB; and CENP-A and grade were assessed by analysis of variance (ANOVA) tests.

To analyze patient outcomes, Kaplan-Meier survival curves [27] for distant relapse-free survival (DRFS) were generated using tertile levels of expression of CENP-A, as there are no published levels of CENP-A expression to use as cutoffs. All cutoff levels were determined prior to data analysis. Survival curves were compared using the log-rank test. For all comparisons, statistical significance was set at *P *< 0.05, and all *P*-values were two-sided.

Univariate and multivariate Cox regression analyses were performed to identify correlations between variables and DRFS up to 5 years for the Wang, TRANSBIG and JBI datasets. Age, estrogen receptor alpha (ESR1) gene expression level, CENP-A gene expression level, Ki-67 gene expression level, and Her2/neu gene expression level were treated as continuous variables. The reference vs. comparison states for categorical variables were, T1 vs. T2 to T4 for tumor stage, 1 to 2 vs. 3 for tumor grade, and negative vs. positive for nodal status. Regression analyses were not performed on the Wang dataset due to the substantial amount of unknown data, or for the ER-negative JBI cohort due to the low number of patients in this group.

Univariate and multivariable logistic regression analyses were performed to identify correlations between variables and chemotherapy response (RCB, as described above) for the MDA233 data set. Predictive variables used were the same as in the DRFS multivariate regression analyses above.

The R statistical environment was used for all calculations [28].

## Results

### CENP-A and CENP-B levels

**Figure 2:**
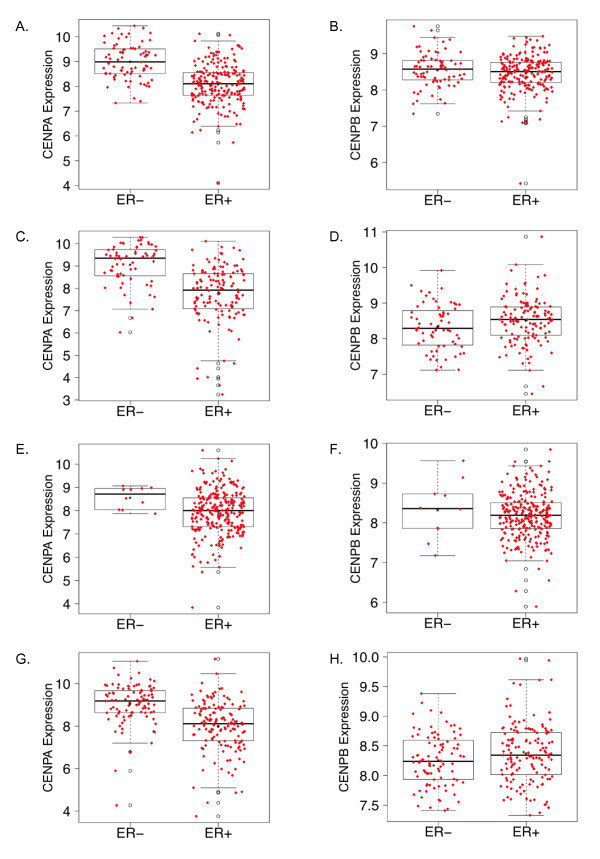
**CENP-A and CENP-B levels in estrogen receptor-negative versus estrogen receptor-positive tumors**. **A**, CENP-A levels in Wang dataset, *P *< 10^-14^. **B**, CENP-B levels in Wang dataset, *P *= 0.26. **C**, CENP-A levels in Transbig dataset, *P *< 10^-12^. **D**, CENP-B levels in Transbig dataset, *P *= 0.06. **E**, CENP-A levels in JBI dataset, *P *= 0.018. **F**, CENP-B levels in JBI dataset, *P *= 0.57. **G**, CENP-A levels in MDA233 dataset, *P *< 10^-12^. **H**, CENP-B levels in MDA233 dataset, *P *= 0.12. CENP-A, centromere protein-A; CENP-B, centromere protein-B.

We began by determining the level of CENP-A in ER-positive versus ER-negative tumors. In the Wang, TRANSBIG, MDA233, and JBI datasets, the levels of CENP-A were significantly higher in ER-negative compared to ER-positive tumors (Figure 2).

We then compared the level of CENP-B in ER-positive versus ER-negative tumors. In all datasets, CENP-B was not different between ER-positive and ER-negative tumors (Figure 2). CENP-B did not correlate with CENP-A when analyzed over all patients and in the subsets of ER-positive and ER-negative patients (data not shown).

Because CENP-A is necessary for centromere specification, higher levels of CENP-A may simply reflect higher levels of cell division. Therefore, we determined the correlation coefficient between CENP-A and Ki-67 in ER-positive and ER-negative tumors (Table 2). In all four datasets of patients with ER-positive and ER-negative disease, CENP-A was positively and significantly correlated with Ki-67. There was minimal or no correlation between CENP-B and Ki-67 (Table 2).

**Table 2:** Correlation of CENP-A and CENP-B with Ki-67 in patients with estrogen receptor-positive and estrogen receptor-negative tumors.

	ER-positive				ER-negative			
	CENP-A		CENP-B		CENP-A		CENP-B	
Dataset	Correlation coefficient vs. Ki-67	*P*-value	Correlation coefficient vs. Ki-67	*P *-value	Correlation coefficient vs. Ki-67	*P *-value	Correlation coefficient vs. Ki-67	*P *-value
Wang	0.718	< 10^-10^	0.158	0.022	0.663	< 10^-10^	0.073	0.525
TRANS	0.576	< 10^-13^	-0.218	0.011	0.574	< 10^-6^	-0.090	0.480
JBI	0.638	< 10^-10^	-0.076	0.219	0.675	0.032	0.216	0.550
MDA233	0.655	< 10^-10^	-0.070	0.404	0.640	< 10^-11^	-0.196	0.063

TRANS, TRANSBIG study population; CENP-A, centromere protein-A; CENP-B, centromere protein-B; ER, estrogen receptor

Similarly, CENP-A may also act as a surrogate marker of grade. In the three datasets containing grade information, CENP-A was positively and significantly correlated with grade for ER-positive cases (Table 3). CENP-A was also positively correlated with grade for ER-negative samples in the TRANSBIG and MDA233 datasets (Table 3). There were only ten patients with ER-negative disease in the JBI dataset, so the analysis was not performed on this subset.

**Table 3:** Correlation of centromere protein-A with grade in patients with estrogen receptor-positive and estrogen receptor-negative tumors.

	ER-positive		ER-negative	
Dataset	Correlation coefficient	*P*-value	Correlation coefficient	*P *-value
TRANSBIG	0.404	0.00023	0.515	< 10^-5^
JBI	0.518	< 10^-14^	---	---
MDA233	0.367	0.00003	0.315	0.03

ER, estrogen receptor

### Distant relapse-free survival

**Figure 3:**
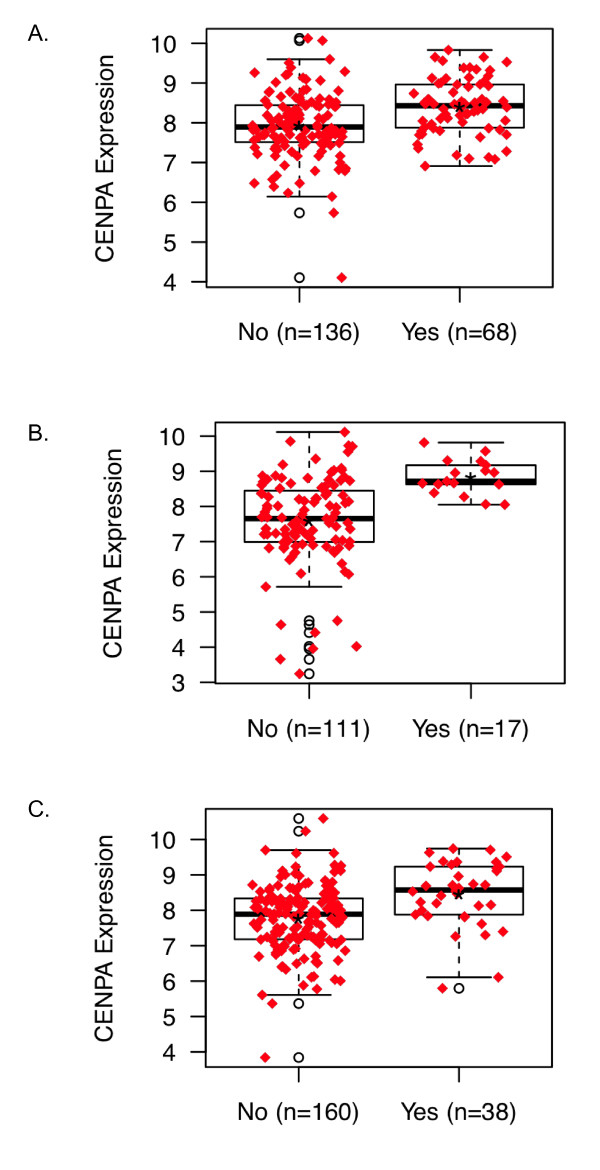
**CENP-A levels in patients with estrogen receptor-positive disease according to distant relapse status at 5 years**. **A**, Wang dataset, *P *< 0.00007. **B**, TRANSBIG dataset, *P *< 0.000009. **C**, JBI dataset, *P *< 0.00003. CENP-A, centromere protein-A.

The median follow-up was 7.2 years in the Wang dataset, 10.0 years in the TRANSBIG dataset, and 6.5 years in the JBI dataset. Among ER-positive patients, CENP-A levels were significantly higher in patients with a distant relapse within five years in the untreated Wang and TRANSBIG cohorts and in the tamoxifen-treated JBI cohort (Figure 3). For ER-negative patients in the Wang and TRANSBIG datasets, there was no difference in CENP-A level between patients with and without a distant relapse at five years (data not shown).

**Figure 4:**
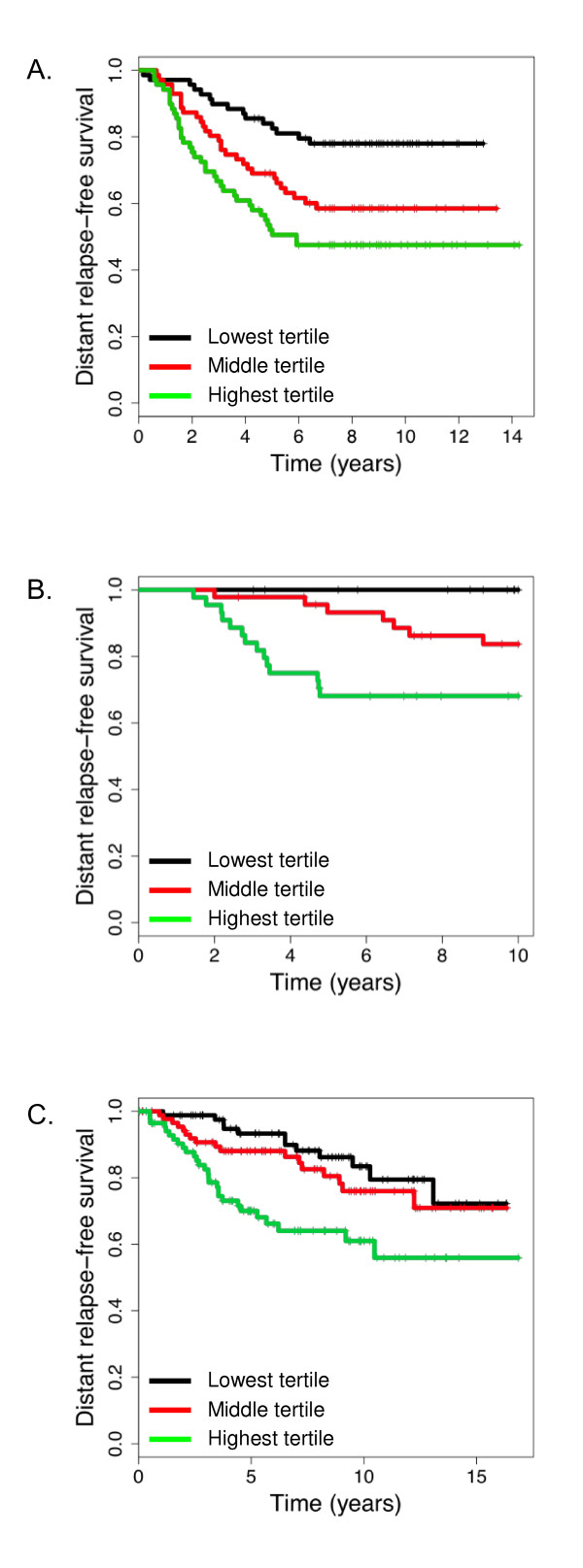
**Distant relapse-free survival in patients with estrogen receptor-positive disease grouped by tertile of CENP-A expression**. In all panels, black line = lowest tertile of CENP-A expression, red line = middle tertile of CENP-A expression, and green line = highest tertile of CENP-A expression. **A**, Wang dataset, *P *= 0.0005. **B**, TRANSBIG dataset, *P *= 0.0001. **C**, JBI dataset, *P *< 0.0008. CENP-A, centromere protein-A.

There are no previously established cutoffs for high or low CENP-A levels, and the distribution of CENP-A levels did not show a clear cutoff in any of the datasets (data not shown). Therefore, patients were grouped by tertile of CENP-A expression. Among patients with ER-positive disease in the untreated Wang and TRANSBIG datasets, higher levels of CENP-A were consistently correlated with decreased DRFS (Figure 4). In the Wang dataset, patients with the highest tertile of CENP-A expression had 5-year DRFS rates of 51% compared to 68% and 83% for patients in the middle and lowest tertiles of CENP-A expression (*P *= 0.0005, Figure 4A). A similar pattern was found for the TRANSBIG dataset (Figure 4B), with 5-year DRFS rates of 68%, 91%, and 100% (*P *= 0.0001). Among patients with ER-positive disease who were treated with tamoxifen in the JBI dataset, patients in the highest tertile of CENP-A expression had 5-year DRFS of 68% vs. 86% and 90% for patients in the middle and lowest tertile (*P *< 0.0008, Figure 4C).

For patients with ER-negative disease in the untreated datasets, there was no difference in 5-year DRFS based on tertile of CENP-A expression. In the Wang dataset, the 5-year DRFS for patients in the highest, middle, and lowest tertile of CENP-A level was 69%, 48%, and 58% respectively (*P *= 0.5). In the TRANSBIG dataset, the 5-year DRFS was 81%, 73%, and 48% for patients in the highest, middle, and lowest tertile (*P *= 0.065).

To determine if CENP-A provides independent prognostic or predictive information for DRFS, univariate and multivariate analyses were performed with age, T stage, tumor grade, and nodal status as categorical variables and with ESR1, CENP-A, Ki-67, and Her2/neu gene expression levels as continuous variables. Among patients with ER-positive disease in the untreated TRANSBIG dataset, univariate analysis revealed that grade, Ki-67, and CENP-A were significantly associated with an increased risk of relapse at 5 years (Table 4). To explore the interaction of grade, Ki-67, and CENP-A, multivariate analyses of the untreated TRANSBIG dataset were performed with and without CENP-A (Table 4). When CENP-A was not included in the multivariate analysis, Ki-67 was significantly associated with DRFS (hazard ratio (HR) 2.83; 95% confidence interval (CI), 1.48 to 5.42; *P *= 0.0017) and grade was borderline significantly correlated with DRFS (HR 2.65; 95% CI, 0.93 to 7.55; *P *= 0.069). When CENP-A was included in the multivariate analysis, neither Ki-67 nor grade correlated with DRFS (Table 4).

**Table 4:** Univariate and multivariate analyses for distant relapse at 5 years in patients with estrogen receptor-positive disease in the TRANSBIG dataset.

	Univariate analyses			Multivariate analysis without CENP-A			Multivariate analysis with CENP-A		
Variable	HR	95% CI	*P*-value	HR	95% CI	*P *-value	HR	95% CI	*P *-value
Age	0.98	0.92-1.05	0.62	1.00	0.93-1.08	0.95	1.01	0.93-1.09	0.89
T stage	1.22	0.47-3.16	0.68	0.82	0.31-2.19	0.70	0.59	0.21-1.67	0.32
Grade	2.70	1.04-7.00	0.04	2.65	0.93-7.55	0.069	1.01	0.33-3.11	0.98
ESR1	1.32	0.87-1.98	0.19	1.63	1.05-2.52	0.025	2.00	1.30-3.08	0.0015
CENP-A	3.30	1.84-5.89	0.000056	---	---	---	10.93	2.86-41.78	0.00047
Ki-67	2.18	1.32-3.60	0.0022	2.83	1.48-5.42	0.0017	1.03	0.58-1.84	0.91
Her2/neu	1.11	0.74-1.66	0.63	1.14	0.79-1.65	0.49	1.10	0.77-1.58	0.60

ESR1 (estrogen receptor alpha), CENP-A (centromere protein-A), Ki-67, and Her2/neu are continuous variables reflecting mRNA levels; HR, hazard ratio; CI, confidence interval; DRFS, distant-relapse free survival; RCB, residual cancer burden

Unexpectedly, ESR1 had a HR of 2.00 (95% CI, 1.30 to 3.08; *P *= 0.0015) for relapse on multivariate analysis of ER-positive tumors in the TRANSBIG dataset (Table 4). On univariate analysis, ESR1 was not associated with an increased risk of relapse (HR = 1.32; 95% CI, 0.87 to 1.98, *P *= 0.19; Table 4). This discrepancy results suggests that the significance of ESR1 seen on multivariate analysis of the TRANSBIG dataset was the result of a high-dimension artifact.

For patients with ER-positive disease in the tamoxifen-treated JBI cohort, CENP-A was significantly associated with 5-year DRFS on univariate analysis (HR 1.97, 95% CI 1.38 to 2.80, *P *= 0.00017) and borderline significant on multivariate analysis (95% CI, 0.99 to 2.71, *P *= 0.054; Table 5). For patients with ER-negative breast cancer in the TRANSBIG dataset, CENP-A was not significantly associated with DRFS on univariate (HR 0.79; 95% CI, 0.51 to 1.23; *P *= 0.30) or multivariate analysis (Table 6). Similarly, for patients with ER-negative disease in the MDA233 dataset, CENP-A was not significant for distant relapse on univariate (HR 1.38; 95% CI, 0.88 to 2.17; *P *= 0.16) or multivariate analysis (Table 6).

**Table 5:** Multivariate analyses for distant relapse in patients with estrogen receptor-positive disease in the JBI and MDA233 datasets.

	JBI (5 y DRFS)			MDA233 (RCB 0/1)		
Variable	HR	95% CI	*P*-value	HR	95% CI	*P *-value
Age	0.99	0.95-1.03	0.48	1.02	0.98-1.06	0.38
T stage	2.09	0.89-4.89	0.09	3.22	0.35-30.01	0.30
Nodal status	0.99	0.47-2.08	0.98	0.39	0.15-1.00	0.05
Grade	0.79	0.31-2.00	0.61	1.09	0.41-2.89	0.86
ESR1	1.00	0.72-1.41	0.98	0.79	0.55-1.12	0.18
CENP-A	1.64	0.99-2.71	0.054	1.30	0.75-2.28	0.35
Ki-67	1.13	0.73-1.77	0.58	1.08	0.64-1.81	0.78
Her2/neu	0.94	0.67-1.31	0.71	1.05	0.67-1.64	0.84

ESR1 (estrogen receptor alpha), CENP-A (centromere protein-A), Ki-67, and Her2/neu are continuous variables reflecting mRNA levels; HR, Hazard ratio; CI, confidence interval; DRFS, distant-relapse free survival; RCB, residual cancer burden

**Table 6:** Multivariate analyses for distant relapse for patients with estrogen receptor-negative disease.

	TRANSBIG (5 y DRFS)			MDA233 (RCB 0/1)		
Variable	HR	95% CI	*P*-value	HR	95% CI	*P *-value
Age	0.99	0.92-1.07	0.84	0.93	0.88-0.98	0.01
T stage	0.81	0.29-2.26	0.69	0.06	0.00-0.82	0.035
Nodal status	---	---	---	0.88	0.25-3.10	0.84
Grade	2.18	0.41-11.67	0.36	2.16	0.52-9.00	0.29
ESR1	0.73	0.48-1.10	0.13	0.82	0.58-1.15	0.26
CENP-A	0.49	0.20-1.20	0.12	1.32	0.65-2.66	0.44
Ki-67	0.63	0.24-1.67	0.36	1.01	0.53-1.93	0.97
Her2/neu	0.90	0.69-1.19	0.46	0.97	0.61-1.54	0.91

ESR1 (estrogen receptor alpha), CENP-A (centromere protein-A), Ki-67, and Her2/neu are continuous variables reflecting mRNA levels; HR, Hazard ratio; CI, confidence interval; DRFS, distant-relapse free survival; RCB, residual cancer burden

### Response to neoadjuvant chemotherapy

**Figure 5:**
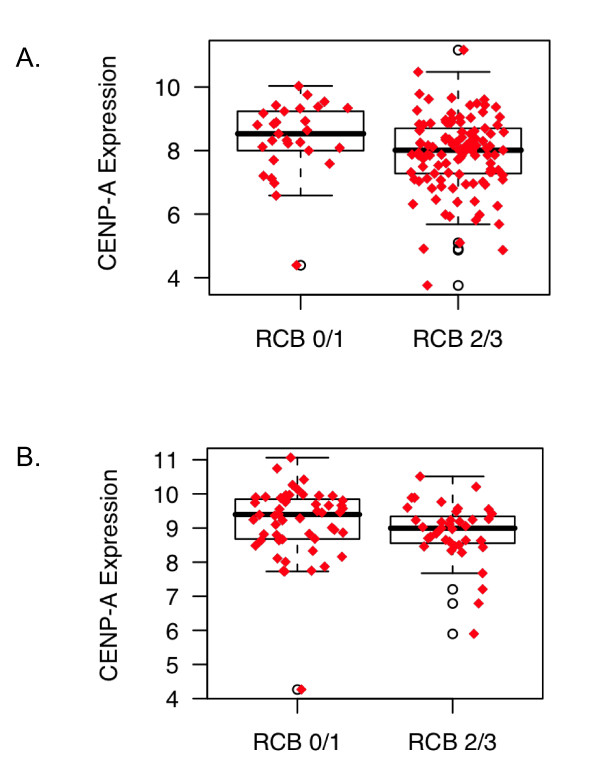
**CENP-A levels in tumors of patients with no or minimal residual disease (RCB 0/1) vs. patients with moderate or extensive residual disease (RCB 2/3) after chemotherapy**. **A**, Patients with estrogen receptor (ER)-positive disease, *P *= 0.0215. **B**, Patients with ER-negative disease, *P *= 0.0433. CENP-A, centromere protein-A.

To determine if CENP-A was associated with response to neoadjuvant chemotherapy, the MDA233 dataset was queried to determine CENP-A levels in patients with no or minimal residual disease after chemotherapy (RCB = 0/1) and in patients with moderate or extensive residual disease after chemotherapy (RCB = 2/3). In patients with ER-positive and ER-negative disease, CENP-A levels were higher in patients with no or minimal residual disease (Figure 5). CENP-B levels were not different between patients in the RCB 0/1 or RCB 2/3 groups for either ER-positive or ER-negative patients (data not shown).

Regression analyses were performed to determine if CENP-A level independently correlated with response to chemotherapy. On multivariate analysis of patients with ER-positive disease in MDA233, nodal status was marginally associated (*P *= 0.05) with RCB 0/1 vs. RCB 2/3 (Table 5). Similar analysis of patients with ER-negative disease in MDA233 identified age and T stage as significantly associated with chemotherapy response (Table 6).

## Discussion

We discovered that CENP-A is an independent prognostic marker for relapse in node-negative, ER-positive breast cancers not treated with systemic therapy. On multivariate analysis, ER-positive patients with elevated CENP-A have a 10.9-fold increased risk of distant relapse within five years in the absence of chemotherapy or hormonal therapy. Furthermore, survival analysis of two independent datasets showed that the level of CENP-A is directly proportional to the risk of distant relapse within five years.

CENP-A correlated with Ki-67 and grade, which have been associated with an increased risk of relapse [29,30]. Our finding that elevated CENP-A was associated with an increased risk of relapse even with the inclusion of Ki-67 and grade in the regression analysis suggests that CENP-A may have a role in recurrence that is independent of its role as a marker of proliferation. Heun, *et al. *have shown that overexpression of CENP-A causes ectopic formation of functional kinetochores and multicentric chromosomes [4]. Similarly, Tomonaga, *et al. *analyzed 11 samples of colorectal cancers and found that CENP-A was overexpressed in all of the samples. In addition to localizing to the centromere, the overexpressed CENP-A also localized to non-centromeric regions of the chromatin [7]. Together, these observations suggest a mechanism for the contribution of CENP-A to aneuploidy and subsequent cancer progression.

Although CENP-A correlates with Ki-67, overexpression of CENP-A independent of the higher proliferation rates of tumor cells is further supported by the observations of Tomonga and colleagues that the level of proliferating cell nuclear antigen (PCNA) was generally similar in tumor and normal cells [7]. Additionally, CENP-B was not correlated with CENP-A in the tumors of patients in our current study, suggesting that elevated levels of CENP-A are not due to increased expression of all centromere proteins. Our observation agrees with a previous observation that CENP-B levels are similar in normal and colorectal tumor samples [7].

Even though CENP-A was elevated in both ER-positive and ER-negative tumors with a complete or near-complete response to neoadjuvant chemotherapy, CENP-A did not independently predict response to chemotherapy. One possible reason is that because CENP-A is proportional to Ki-67, which has predictive value for chemotherapy response [31], CENP-A did not provide additional predictive power for chemotherapy response. However, given the relatively modest sample size, a small effect cannot be ruled out.

We found that ER-negative tumors had higher levels of CENP-A than ER-positive tumors. This result is consistent with several previous studies that have shown that ER-negative breast cancer has a distinct genetic signature from ER-positive disease [32-34]. Higher levels of CENP-A in ER-negative tumors may reflect a higher rate of proliferation or chromosomal instability [35,36] compared to ER-positive cancers.

Consistent with our results, Hu, *et al. *recently found that Holliday Junction Recognition Protein (HJURP), which has been proposed to act as a CENP-A-specific chaperone responsible for the deposition of CENP-A at centromeres [37,38], is also an independent prognostic marker in breast cancer [39]. They showed that HJURP levels were higher in ER-negative tumors and were strongly correlated with Ki-67 and CENP-A levels. Higher levels of HJURP were associated with decreased survival, and on multivariate analysis for disease-free survival, HJRUP had a HR of 2.05 [39]. Notably, this multivariate analysis did not include Ki-67 or CENP-A, which may have accounted for some of the variation in survival. Clearly, future studies on the interactions of HJRUP and CENP-A in breast cancer are warranted.

Although CENP-A levels were higher in ER-negative tumors, CENP-A was only prognostic for DRFS in patients with ER-positive tumors. This is consistent with both models of estrogen modulation of CENP-A described above. In the FOXM1 mechanism, increased ER signaling would increase levels of FOXM1, resulting in increased levels of CENP-A. In agreement with this, recent work has shown that FOXM1 is an independent prognostic marker in ER-positive but not ER-negative breast cancer [40]. In the Aurora A kinase model, estrogen drives overexpression of Aurora A kinase [14], which is required for phosphorylation of CENP-A and subsequent kinetochore function [15]. It is possible that the presence of elevated levels of both CENP-A and Aurora A in ER-positive tumors would portend a worse outcome than the presence of increased levels of CENP-A alone. Estrogen-driven expression of Aurora A would be absent from ER-negative tumors, which might account for the lack of prognostic utility of CENP-A in ER-negative tumors.

In the tamoxifen-treated JBI dataset, CENP-A was higher in patients with a distant relapse, and the level of CENP-A was directly proportional to the risk of relapse. The borderline significance of CENP-A on multivariate analysis for DRFS in the JBI dataset most likely reflects the strong prognostic role of CENP-A in ER-positive patients as seen in the Wang and TRANSBIG cohorts that did not receive systemic therapy. Alternatively, it is possible that treatment with tamoxifen blocks estrogen-driven modulation of CENP-A via either the FOXM1 or Aurora A pathways, minimizing the influence of estrogen on CENP-A levels. Future studies on the influence of tamoxifen and other hormonal therapies on mitosis are plainly needed.

One weakness of this study is that well-known clinical elements such as T stage and nodal status were not robustly identified as prognostic factors across all four datasets. This is likely the result of the variable manner in which samples were chosen for each dataset. For instance, samples included in the Wang dataset were retrospectively chosen from technically satisfactory samples available from patients with good, intermediate, and poor outcomes [18]. Moreover, 87% of these patients received radiation, which may have affected their survival. Ideally, future biomarker studies will utilize samples collected prospectively from patients treated in a uniform fashion.

## Conclusions

We found CENP-A to be a strong prognostic marker for distant relapse in ER-positive breast cancer. Even when known clinical factors such as Ki-67 and grade are considered, CENP-A remains an independent prognostic marker for relapse, suggesting that CENP-A may contribute to disease progression independent of its role as a marker of proliferation. Moreover, the level of CENP-A was directly proportional to the risk of distant relapse, demonstrating a clear relationship between the degree of expression of this essential protein and outcome in ER-positive breast cancer.

## Abbreviations

CENP-A: centromere protein-A; CENP-B: centromere protein-B; CI: confidence interval; DRFS: distant relapse free survival; ER: estrogen receptor; ESR1: estrogen receptor alpha; FOXM1: forkhead box protein M1; GEO: Gene Expression Omnibus; HJURP: Holliday Junction Recognition Protein; HR: hazard ratio; JBI: Institut Jules Bordet; PR: progesterone receptor; RCB: residual cancer burden; T/FAC: paclitaxel, 5-fluorouracil, doxorubin, and cyclophosphamide.

## Competing interests

The authors declare that they have no competing interests.

## Authors' contributions

SLM conceived the study, participated in its design, and drafted the manuscript. YQ performed the statistical analysis. LP, WFS, and TAB participated in the study design and helped to draft the manuscript. All authors read and approved the final manuscript.
